# Supplementary material for: Integrating tough Antheraea pernyi silk and strong carbon fibres for impact-critical structural composites
Source: Nat Commun. 2019 Aug 22;10:3786. doi: 10.1038/s41467-019-11520-2 (PMC6706406; doi:10.1038/s41467-019-11520-2)
Supplement: Supplementary file 1 — Supplementary Information [file 41467_2019_11520_MOESM1_ESM.pdf]

Supplementary Information

Integrating tough *Antheraea pernyi* silk and strong carbon fibres for impact-critical structural composites

Kang Yang, Juan Guan, Keiji Numata, Change Wu, Sujun Wu,
Zhengzhong Shao, and Robert O. Ritchie

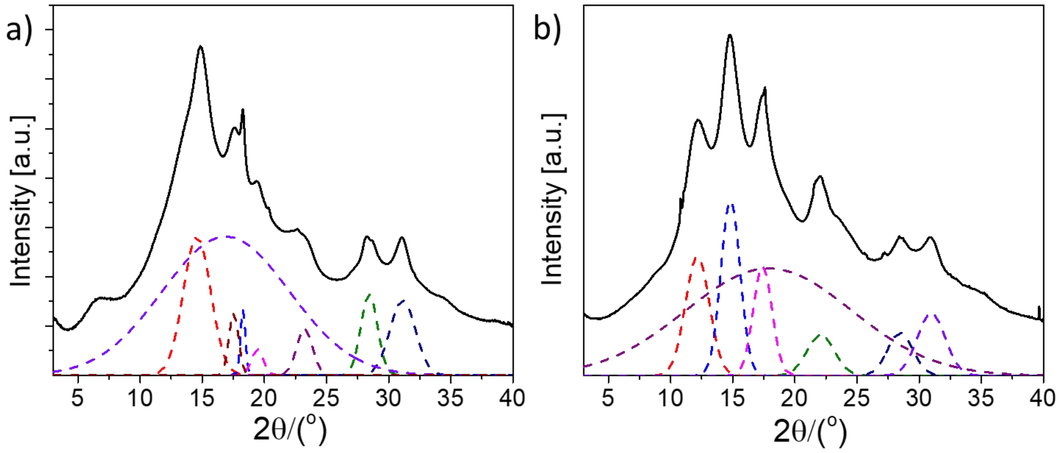

Supplementary Figure 1 Peak-fitting analysis of the WAXS profiles using Gaussian functions. **a** *B.mori* silk, **b** *A. pernyi* silk.

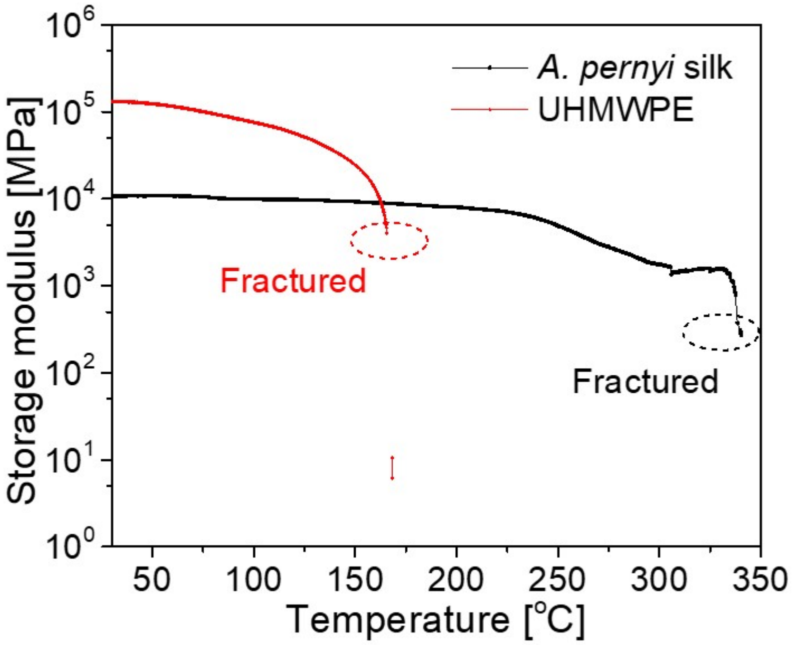

Supplementary Figure 2 DMTA plots of storage modulus and *tanδ* for natural *A. pernyi* silk and UHMWPE.

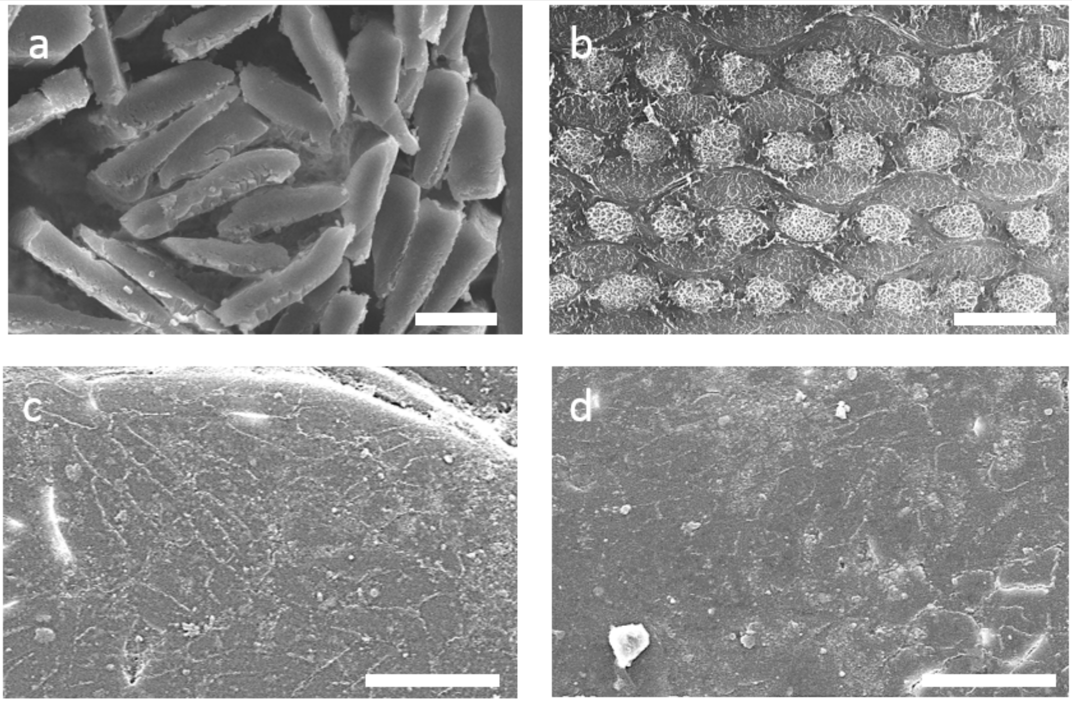

Supplementary Figure 3 SEM images of the cross-sectional morphologies. **a**. *pernyi* silk bundle, and **b**, **c** and **d** SFRP at various magnifications. Scale bars: **a** 20 μm, **b** 500 μm, **c** and **d** 50 μm.

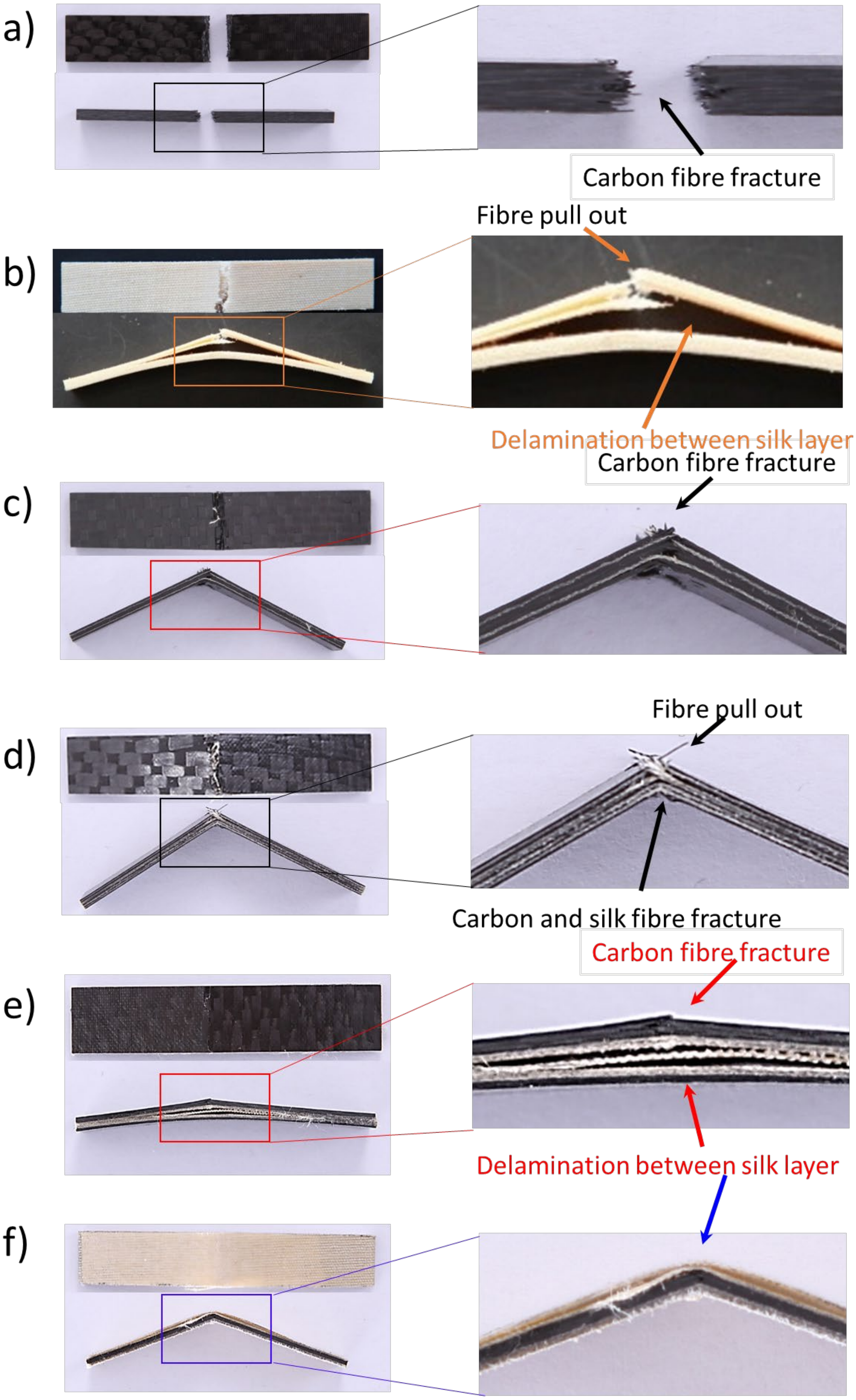

Supplementary Figure 4 Fracture morphology of different composites after unnotched Charpy impact testing. **a** CFRP, **b** SFRP, **c** 8C2S, **d** 5C5S-1, **e** 5C5S-2, and **f** 5C5S-3.

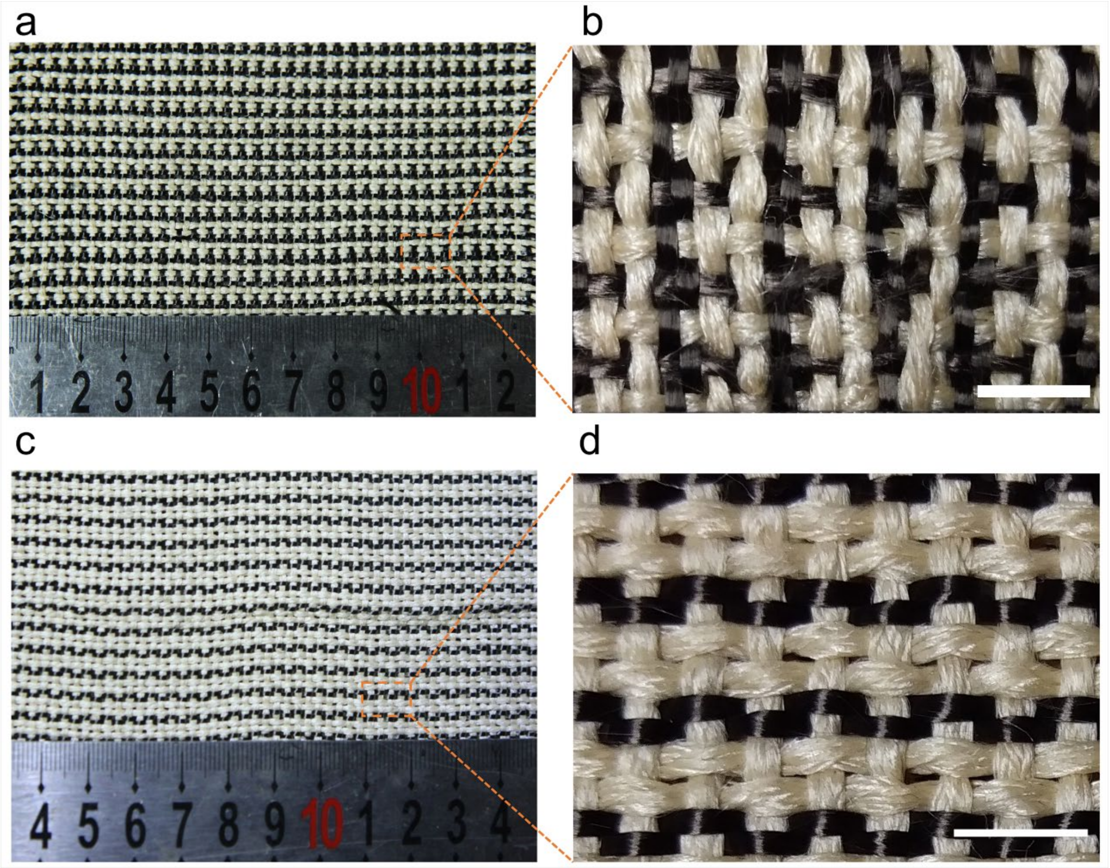

Supplementary Figure 5 Photographs of two intra-ply/IP hybrid fabrics from *A. pernyi* silks and carbon fibres. **a** and **b** IP-1 and **c** and **d** IP-2. Note that the two fabrics were designed and woven at a textile factory. Scale bars: **b** and **d** 5 mm.

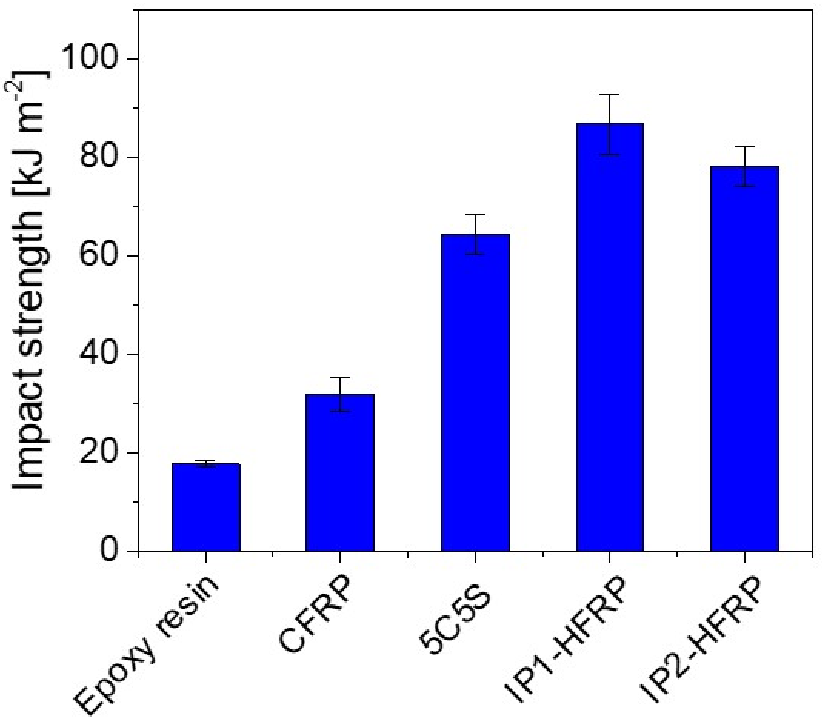

Supplementary Figure 6 Charpy impact strength of different samples. The samples include epoxy resin matrix, CFRP, inter-ply HFRP/5C5S and HFRPs from the two intra-ply hybrid fabrics, IP1-HFRP and IP2-HFRP. Note that the epoxy resin matrix was epoxy 1564 with hardeners 3486. The error bars represented the standard deviation of measured means.

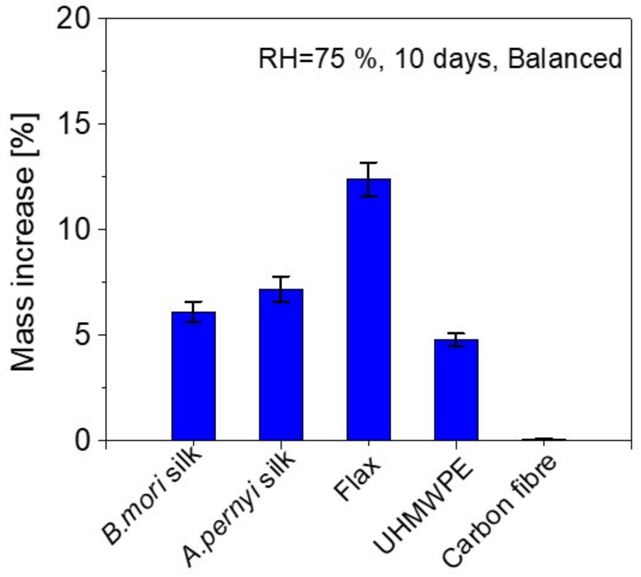

Supplementary Figure 7 Comparison of the mass increase of five fibres after environmental conditioning. The error bars represented the standard deviation of measured means.

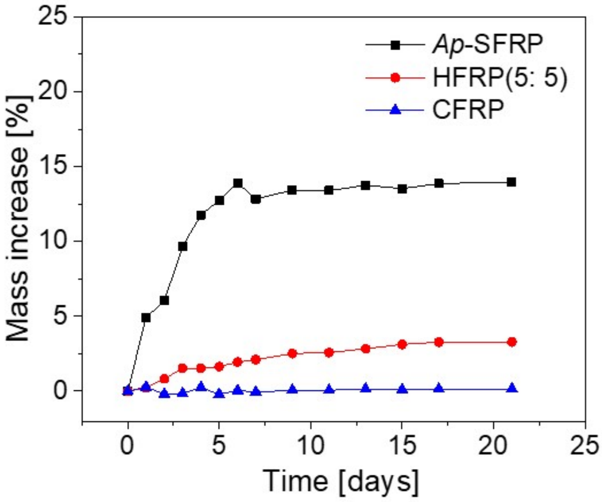

Supplementary Figure 8 Comparison of mass increase of different composites as a function of immersion time. The immersion condition was in ultrapure water at 23ºC ± 2ºC. Note that the epoxy resin matrix was epoxy 1564 with hardeners 3486.

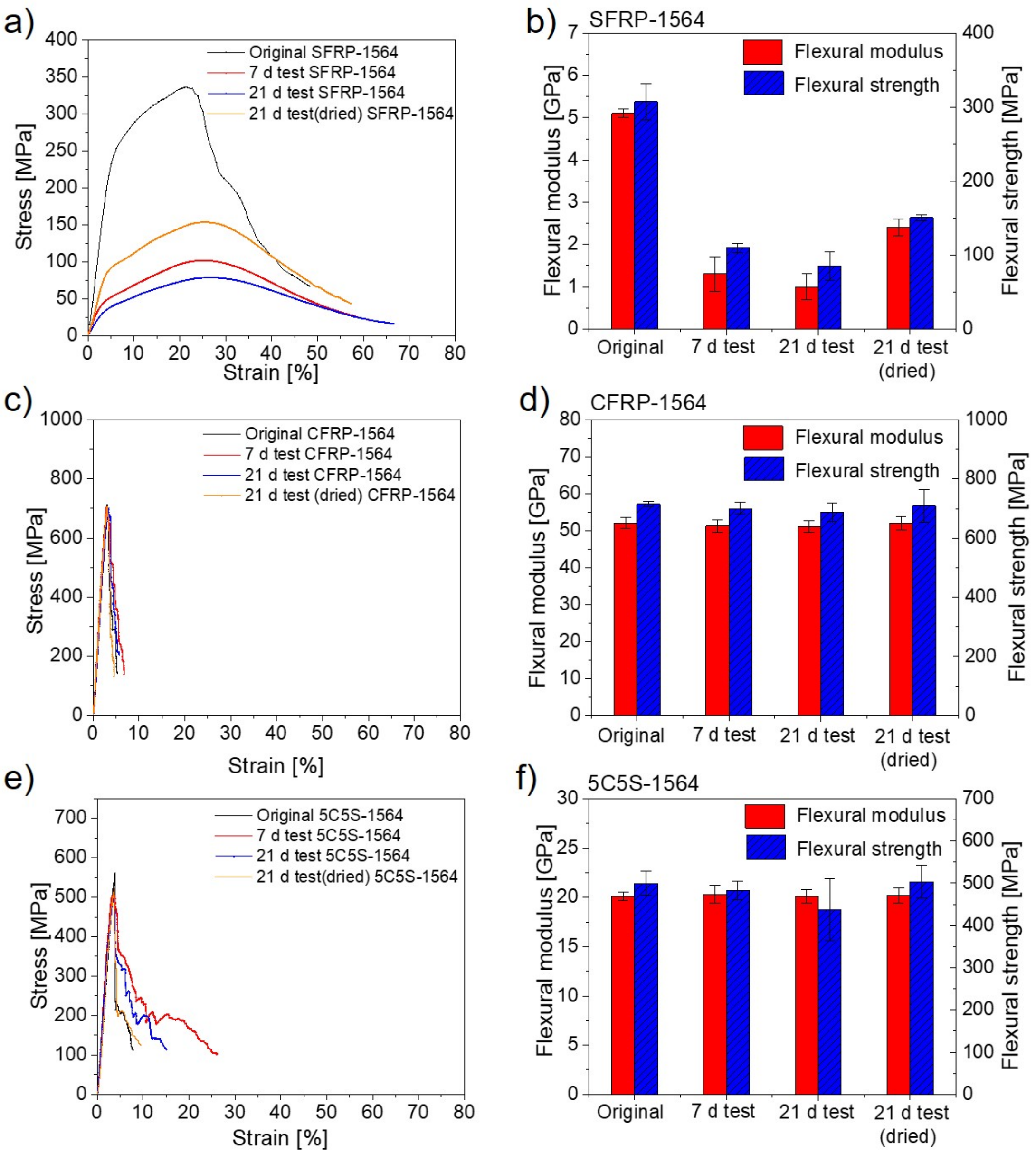

Supplementary Figure 9 Comparison of flexural properties of three composites. **a** and **b** SFRP, **c** and **d** CFRP, **e** and **f** HFRP/5C5S, before and after the water-immersion treatment for 7 days and 21 days. Note: The epoxy resin matrix was epoxy 1564 with hardeners 3486. The error bars represented the standard deviation of measured means.

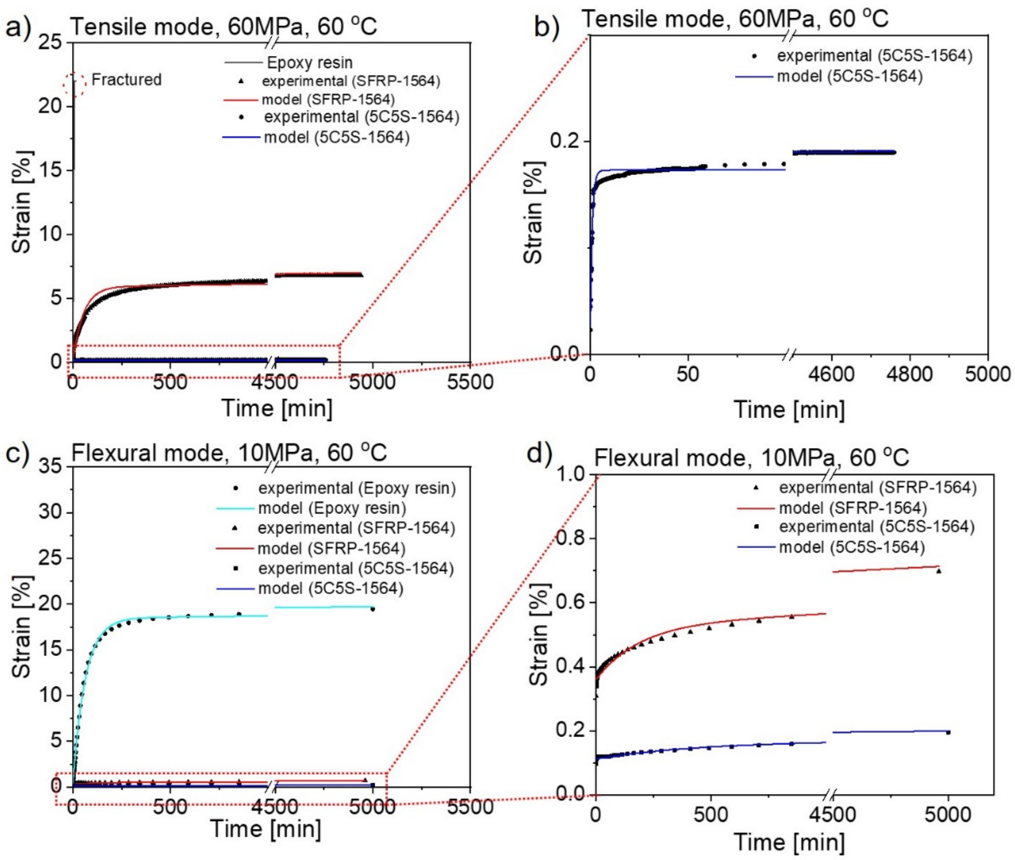

Supplementary Figure 10 Creep behaviour of epoxy resin, *A. pernyi* SFRP and HFRP/5C5S. **a** and **b** tensile stress of 60 MPa, and **c** and **d** flexural stress of 10 MPa. Note that the epoxy resin matrix was epoxy 1564 with hardeners 3486.

Supplementary Table 1 Mechanical properties of the resin and composites in this work.

**Specimen**	**Physical properties**		**Tensile properties**						**Flexural properties**				**Impact properties**	
	ρ	V_f	E_t	E_t/ρ	σ_t	σ_t/ρ	ε_t	BE_t	E_f	E_f/ρ	σ_f	σ_f/ρ	σ_i	σ_i/ρ
Epoxy resin	1.2	0	3.2±0.1	2.7±0.1	76.6±1.3	63.8±1.1	2.8±0.1	1.1±0.1	3.5±0.0	2.9±0.0	134.2±6.4	111.8±5.3	12.8±0.2	10.7±0.1
CFRP	1.61	69	65.2±3.2	40.5±2.0	554.8±13.7	344.6±8.5	0.91±0.0	2.4±0.1	55.1±2.1	34.2±1.3	1002.1±28.5	622.4±17.7	43.3±3.7	26.9±2.3
8C2S	1.56	65	63.5±3.8	40.7±2.4	501.4±14.2	321.4±9.1	0.86±0.1	2.4±0.2	43.2±2.5	27.7±1.6	571.3±17.4	366.2±11.2	73.6±5.2	47.2±3.3
5C5S-1	1.43	61	39.3±2.7	27.4±1.9	380.0±10.9	265.7±7.6	1.0±0.1	2.0±0.1	38.1±2.4	26.6±1.7	641.3±25.3	448.5±17.7	97.5±4.7	68.2±3.3
5C5S-2	1.43	61	38.5±3.0	26.9±2.1	376.5±8.9	263.3±6.2	0.99±0.1	2.0±0.1	15.2±0.8	10.6±0.6	461.2±18.9	322.5±13.2	85.5±4.8	59.8±3.4
5C5S-3	1.43	61	40.6±2.8	28.3±1.9	393.3±10.4	275.0±7.3	1.1±0.0	2.0±0.1	34.4±2.8	24.0±1.9	335.9±21.4	234.9±15.0	80.4±6.2	56.2±4.3
2C8S	1.32	56	17.2±1.7	13.0±1.3	176.0±5.4	133.3±4.1	1.2±0.1	1.2±0.2	31.8±2.6	24.1±2.0	262.6±17.3	198.9±13.1	77.3±6.0	58.6±4.5
SFRP	1.25	51	7.8±0.2	6.2±0.2	129.3±3.2	103.4±2.6	9.6±0.1	9.9±0.3	6.8±0.3	5.4±0.2	256.6±13.6	205.3±10.9	90.8±5.4	72.6±4.3

ρ:density (10^3 kg.m^{-3}), V_f: fibre volume fraction (%), E_t: tensile modulus (GPa), ρ:density, E_t/ρ: specific tensile modulus (GPa(10^3 kg.m^{-3})$^{-1}$), σ_t: tensile strength (MPa), σ_t/ρ: specific tensile strength (MPa(10^3 kg.m^{-3})$^{-1}$), ε_t: ultimate tensile strain (%), BE_t: tensile fracture energy (MJ.m^{-3}), E_f: flexural modulus (GPa), E_f/ρ: specific flexural modulus (GPa(10^3 kg.m^{-3})$^{-1}$), σ_f: flexural strength (MPa), σ_f/ρ: specific flexural strength (MPa(10^3 kg.m^{-3})$^{-1}$), σ_i: impact strength (kJ.m^{-2}), σ_i/ρ: specific impact strength (kJ.m^{-2}(10^3 kg.m^{-3})$^{-1}$)

Supplementary Table 2 Dataset of density and impact strength of the composites in the literature.

**Sample**	**Volume fraction**	**Density [10^3 kg m^{-3}]**	**Impact strength [kJ m^{-2}]**	**Reference**
Flax-PP	20%	1.02	10	1
Flax-PP	40%	1.11	15	1
Flax-epoxy	35%	1.27	10.5 ± 1.1	2
Hemp-epoxy	65%	1.37	12	3
Hemp-PLA	30%	1.28	9	4
Kenaf-PLA	40%	1.32	14	5
Hemp-PLA	30%	1.28	19	6
Kenaf-PHB	40%	1.35	10	5
Jute-Acrylic	None	1.25	8.8 ± 1.0	7
Jute-polyester	None	1.27	10.6 ± 1.0	7
Sisal-acrylic	None	1.20	12.7 ± 1.4	7
Sisal-polyester	None	1.19	12.2 ± 1.7	7
Flax-acrylic	None	1.22	15.0 ± 0.9	7
Flax-polyester	None	1.21	13.2 ± 0.9	7
Hemp-PLA	45%	1.32	25	8
PALF-polyester	30%	1.27	24	9
Flax-PP	30%	1.06	22	10
Flax-PP	30%	1.06	18	11
Carbon-epoxy	66%	1.6	109.8	12
Carbon-polyimide	30%	1.49	11.5	13
Carbon-epoxy	10%	1.26	19.6	14
Carbon-epoxy	None	None	69.4	15
Carbon-epoxy	40%	1.44	27.8	16
Carbon-epoxy	55%	1.53	114	17
Glass-epoxy	None	1.69	168	18
Glass-PP	35%	1.51	100	19
Glass-Acrylic	30%	1.62	98.7	20
Glass-Polyester	30%	1.62	106.5	20
Glass-epoxy	30%	1.62	122	21
Glass-acrylic	None	1.71	98.7 ± 8.0	7
Glass-polyester	None	1.64	106.5 ± 4.2	7
Glass-polyester	57%	2.02	69	22
Glass-epoxy	61%	2.05	137	22

N.B., We estimated the density in ref. 15 as 1.5×10^3 kg m^{-3} from the matrix and fibre species.

Supplementary References

1. Van den Oever, M. J. A., Bos, H. L. & Molenveld, K. Flax fibre physical structure and its effect on composite properties: Impact strength and thermo-mechanical properties, *Die Angew. Makromol. Chem*. **272**, 71-76 (1999).
2. George, J., Weyenberg, I. V. D., Ivens, J. & Verpoest, I. Mechanical properties of flax fibre reinforced epoxy composites. *Macromol. Mater. Eng.* **272**, 41-45(1999).
3. Islam, M. S., Pickering, K. L. & Foreman, N. J. Influence of alkali fiber treatment and fiber processing on the mechanical properties of hemp/epoxy composites. *J. Appl. Polym. Sci.* **119**, 3696-3707(2011).
4. Islam, M. S., Pickering, K. L. & Foreman, N. J. Influence of alkali treatment on the interfacial and physico-mechanical properties of industrial hemp fibre reinforced polylactic acid composites. *Compos. Part A: Appl. Sci. Manufac.* **41**, 596-603(2010).
5. Graupner, N. & Mussig, J. A comparison of the mechanical characteristics of kenaf and lyocell fibre reinforced poly(lactic acid) (PLA) and poly(3-hydroxybutyrate) (PHB) composites. *Compos. Part A: Appl. Sci. Manufac.* **42**, 2010-2019(2011).
6. Baghaei, B. et al. Novel aligned hemp fibre reinforcement for structural biocomposites: Porosity, water absorption, mechanical performances and viscoelastic behavior. *Compos. Part A: Appl. Sci. Manufac.* **61**, 1-12(2014).
7. Rodriguez, E., Petrucci, R., Puglia, D., Kenny, J.M. & Vazquez, A. Characterization of composites based on natural and glass fibers obtained by vacuum infusion. *J. Compos. Mater.* **39**, 265-282(2005).
8. Baghaei, B., Skrifvars, M. & Berglin, L. Manufacture and characterisation of thermoplastic composites made from PLA/hemp co-wrapped hybrid yarn prepregs. *Compos. Part A: Appl. Sci. Manufac.* **50**, 93–101(2013).
9. Devi, L. U., Bhagawan, S. S. & Thomas, S. Mechanical properties of pineapple leaf fiber-reinforced polyester composites. *J. Appl. Polym. Sci.* **64**, 1739-1748(1997).
10. Snijder, M. H. B. & Bos, H. L. Reinforcement of polypropylene by annual plant fibers: optimisation of the coupling agent efficiency. *Compos. Interface* **7**, 69-79(2000).
11. Hill, C. A. S., Khalil, H. P. S. & Hale, M. D. A study of the potential of acetylation to improve the properties of plant fibres. *Ind. Crop. Prod.* **8**, 53-63(1998).
12. Ouyang, G. E., Sun, Q. H. & Lin, F. S. Studying impact behavior of carbon fiber/epoxy composites, fiber reinforced plastics. *Composites*, **4**, 6-7(2003).
13. Wang, X. D., Wang, X., Zhu, P. & Huang, P. Mechanical properties and tribological performance of plastic polyimide reinforced by carbon fibers. *Mat. Sci. Eng. R.* **29**, 42-45(2005).
14. Chang, L., Zhang, Z. & Breidt, C. Impact resistance of short fibre/particle reinforced epoxy. *Appl. Compos. Mater.* **11**, 1-15(2004).

15. Dong, J. D. et al. Improved mechanical properties of carbon fiber-reinforced epoxy composites by growing carbon black on carbon fiber surface. *Compos. Sci. Technol.* **149**, 75-80 (2017).
16. Zhong, W. Z., Ye, F. T. & Yi, T. U. Mechanical properties of epoxy resin composites reinforced by short carbon fibers treated with ball milling-liquid phase oxidation method, *Acta Armamentarii* **34**, 869-875(2013).
17. Adams, D. F. & Miller, A. K. An analysis of the impact behavior of hybrid composite materials. *Mat. Sci. Eng.* **19**, 245-260(1975).
18. Sebastian, H., Tim, W., Lozoya, V. & Tomas, J. Comparison of impact behaviour of glass, carbon and Dyneema composites. *P. I. Mech. Eng. C-J. Mec.* **233**, 951-966(2019).
19. Zhao, N., Rödel, H., Herzberg, C., Gao, S. L. & Krzywinski, S. Stitched glass/PP composite. Part I: Tensile and impact properties. *Compos. Part A: Appl. Sci. Manufac.* **40**, 635-643(2009).
20. Rodriguez, E. Characterization of composites based on natural and glass fibers obtained by vacuum infusion. *J. Compos. Mater.* **39**, 265-82(2005).
21. Aldulaim, Q. Effect of water absorption on impact strength for epoxy-glass fibers composite, *J. Univ. Anbar Pure Sci.* **5**, 1-6, (2011).
22. Yeung, P. & Broutman, L. J. The effect of glass-resin interface strength on the impact strength of fiber reinforced plastics. *Polym. Eng. Sci.* **18**, 62-72(2010).
